# Molecular dissection of HBV evasion from restriction factor tetherin: A new perspective for antiviral cell therapy

**DOI:** 10.18632/oncotarget.4808

**Published:** 2015-08-27

**Authors:** Kei Miyakawa, Satoko Matsunaga, Koichi Watashi, Masaya Sugiyama, Hirokazu Kimura, Naoki Yamamoto, Masashi Mizokami, Takaji Wakita, Akihide Ryo

**Affiliations:** ^1^ Department of Microbiology, Yokohama City University School of Medicine, Kanagawa 236–0004, Japan; ^2^ Department of Virology II, National Institute of Infectious Diseases, Tokyo 162–8640, Japan; ^3^ Department of Hepatic Diseases, The Research Center for Hepatitis and Immunology, National Center for Global Health and Medicine, Chiba 272–8516, Japan; ^4^ Infectious Disease Surveillance Center, National Institute of Infectious Diseases, Tokyo 208–0011, Japan; ^5^ Department of Microbiology, National University of Singapore, Singapore 117597, Singapore

**Keywords:** hepatic injury, pyroptosis, Immunology and Microbiology Section, Immune response, Immunity

## Abstract

Viruses have evolved various strategies to escape from the innate cellular mechanisms inhibiting viral replication and spread. Extensive evidence has highlighted the ineffectiveness of interferon (IFN) therapy against chronic hepatitis B virus (HBV) infection, implying the existence of mechanisms by which HBV evades IFN-induced antiviral responses. In our current study, we demonstrate that HBV surface protein (HBs) plays a crucial role in counteracting the IFN-induced antiviral response mediated by tetherin (also known as BST-2). The type I IFN treatment of HBV-producing cells marginally but significantly inhibited the release of HBsAg and viral DNA, but this release was recovered by the knockdown of tetherin. HBs can interact with tetherin via its fourth transmembrane domain thereby inhibiting its dimerization and antiviral activity. The expression of a tetherin mutant devoid of the HBs-binding domain promoted a prominent restriction of HBV particle production that eventually resulted in the alleviation of caspase-1-mediated cytotoxicity and interleukin-1β secretion in induced pluripotent stem cell (iPSC)-derived hepatocytes. Our current results thus reveal a previously undescribed molecular link between HBV and tetherin during the course of an IFN-induced antiviral response. In addition, strategies to augment the antiviral activity of tetherin by impeding tetherin-HBs interactions may be viable as a therapeutic intervention against HBV.

## INTRODUCTION

The type I interferon (IFN) system, which includes IFNα and IFNβ, is an innate immune response [1]. Upon virus infection, cells can readily secrete IFNα/β as part of the biological defense mechanisms that plays a primary role in virus restriction. Indeed, IFNα/β induces the synthesis of a range of antiviral proteins, which serve as cell-autonomous intrinsic restriction factors [2]. However, viruses have evolved multiple strategies to evade the IFN system, which would otherwise limit viral spread at an early stage of infection [3].

Hepatitis B is a serious infectious illness of the liver caused by the hepatitis B virus (HBV) [4]. The primary treatment goal for patients with hepatitis B is to prevent progression of the disease to cirrhosis, liver failure, or hepatocellular carcinoma. Current antiviral therapies for HBV infection involve nucleoside reverse transcriptase inhibitors (NRTIs). However, the long-term treatment of hepatitis B with NRTIs can be associated with toxicity and the emergence of drug resistant viral mutations, which result in treatment failure and disease progression. Therefore, it is vital to develop a new type of antiviral drugs for hepatitis B treatment. Pegylated IFNα is also the standard first-line agent in the treatment of hepatitis B. The biological response to IFN is mediated by its binding to the IFN receptors and the activation of the Janus-activated kinase–signal transducer and activator of transcription (STAT) pathway. This leads to the expression of several hundred IFN-stimulated genes (ISGs), such as tetherin (also known as BST-2).

Tetherin inhibits the release of HIV, Ebola, Lassa, herpes and other enveloped viruses from infected cells by tethering progeny virions to the membranes of the host cells [5]. However, many viral proteins can inactivate tetherin in multiple ways [6]. For example, HIV-1 Vpu can displace tetherin from the site of viral assembly in the plasma membrane [7, 8]. Ebola virus glycoprotein (GP) can bind tetherin directly for antagonizing its function, although the mechanism was not deciphered [9]. Furthermore, tetherin can induce NF-κB-mediated signal transduction, leading to the production of proinflammatory cytokines, thereby acting as an innate sensor of viral release [10–12].

Accumulating evidence now strongly indicates that IFNα may not be an effective treatment for hepatitis B virus infection [13]. These findings suggest that HBV has evolved strategies to block IFN signal transduction and its antiviral properties. Previous reports indicate that HBV polymerase can block STAT activation to limit IFNα–induced antiviral responses [14, 15]. Although the aforementioned pathway might be associated with a high incidence of resistance to type I IFN in patients with HBV infection, it remains elusive as to whether there are other mechanisms that contribute to the IFN-resistance of HBV in connection with ISGs. Thus, it is likely that HBV has the countermeasures to repress the innate antiviral function of tetherin.

In our present study, we reveal that IFN-induced tetherin can repress the release of HBV from infected cells but is antagonized by the viral protein HBs. We also suggest that the transduction of HBs-resistant tetherin in hepatocytes may be a potential option in the treatment of HBV infection.

## RESULTS

### Type I IFN-induced tetherin marginally represses HBV release

**Figure 1:**
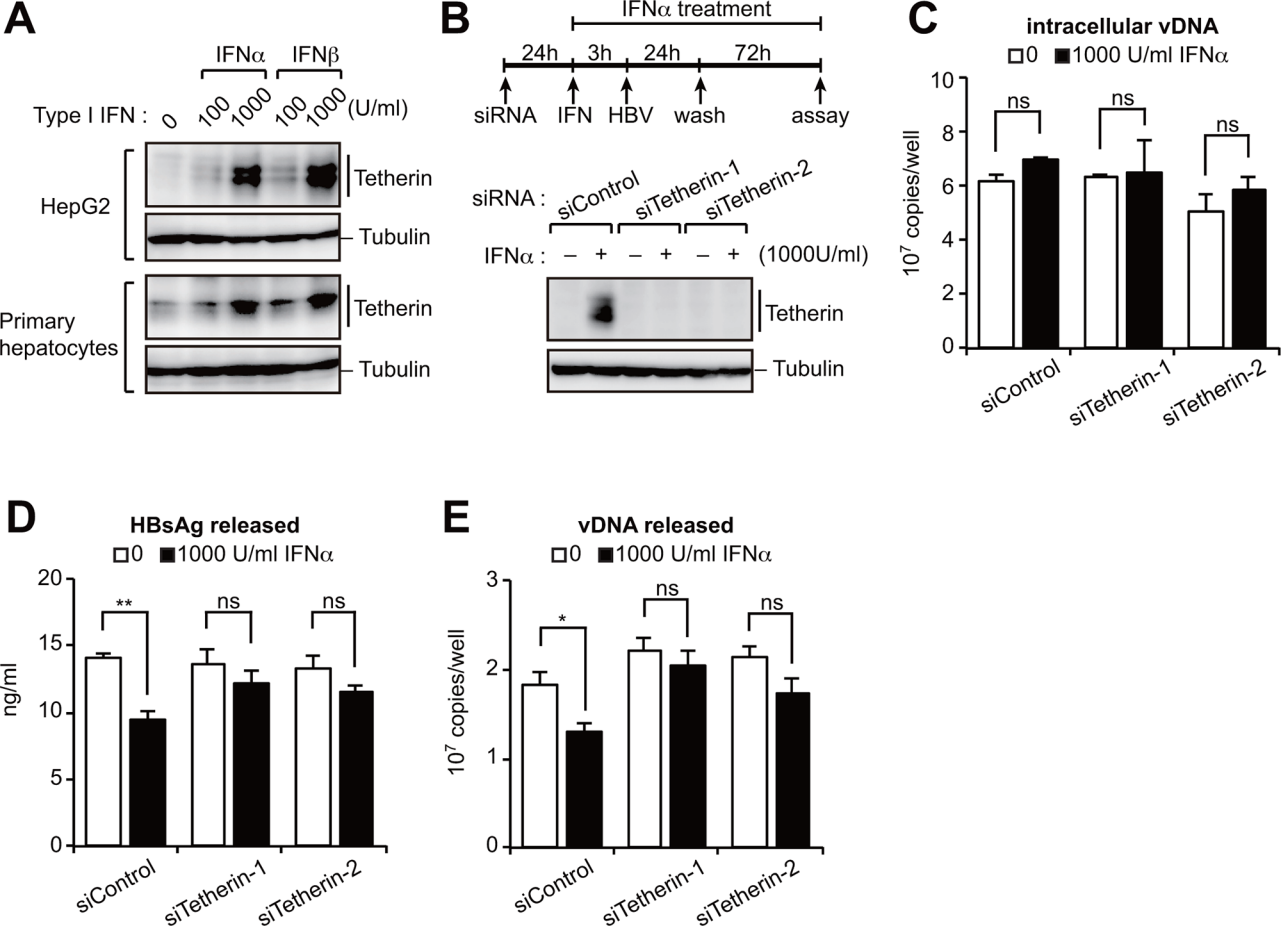
Type I IFN-induced tetherin weakly represses HBV release **A.** Immunoblotting analysis of HepG2 cells (top) and primary hepatocytes (bottom) treated with either IFNα or IFNβ (100 or 1,000 U/ml) for 24 h before harvesting. **B–E.** HepG2 cells were transduced with siRNA targeting tetherin (siTetherin-1 or 2) or control siRNA, and were treated with IFNα, then transfected with an HBV molecular clone (pUC19-C_JPNAT) 24 h later. One day after transfection, the cells were washed and treated with IFNα for three days. The expression of tetherin and tubulin in cells was detected by immunoblotting (B). The amounts of viral DNA (vDNA) in cells (C) and in the culture supernatants **(E)** were measured by real-time PCR. The amounts of HBsAg in the culture supernatants were measured by ELISA (D). ns, not significant; *P < 0.05; **P < 0.01.

Since tetherin is one of many ISGs, we first investigated whether IFNα/β could induce the expression of tetherin in human hepatocytes. Whilst we found a relatively low level expression of tetherin in untreated cells, treatment with type I IFNs increased the tetherin protein to the certain levels in HepG2 cells and primary human hepatocytes (Figure 1A). We then investigated the antiviral response by tetherin induced by IFNα treatment. HepG2 cells were treated with or without tetherin-specific siRNAs and IFNα, and then transfected with a HBV molecular clone (pUC19-C_JPNAT; genotype C) (Figure 1B). Although IFNα did not affect the amounts of intracellular viral DNA (vDNA) (Figure 1C), we found that IFNα weakly but statistically significantly decreased the levels of HBV surface antigen (HBsAg) and vDNA in the culture supernatant (Figure 1D, 1E). Moreover, these IFN-induced effects were reduced by a knockdown of tetherin by siRNA (Figure 1D, 1E). These results indicate that IFN-induced tetherin acts as an antiviral effector against HBV release, although in a relatively weak manner.

### HBs binds tetherin via its fourth transmembrane domain

**Figure 2:**
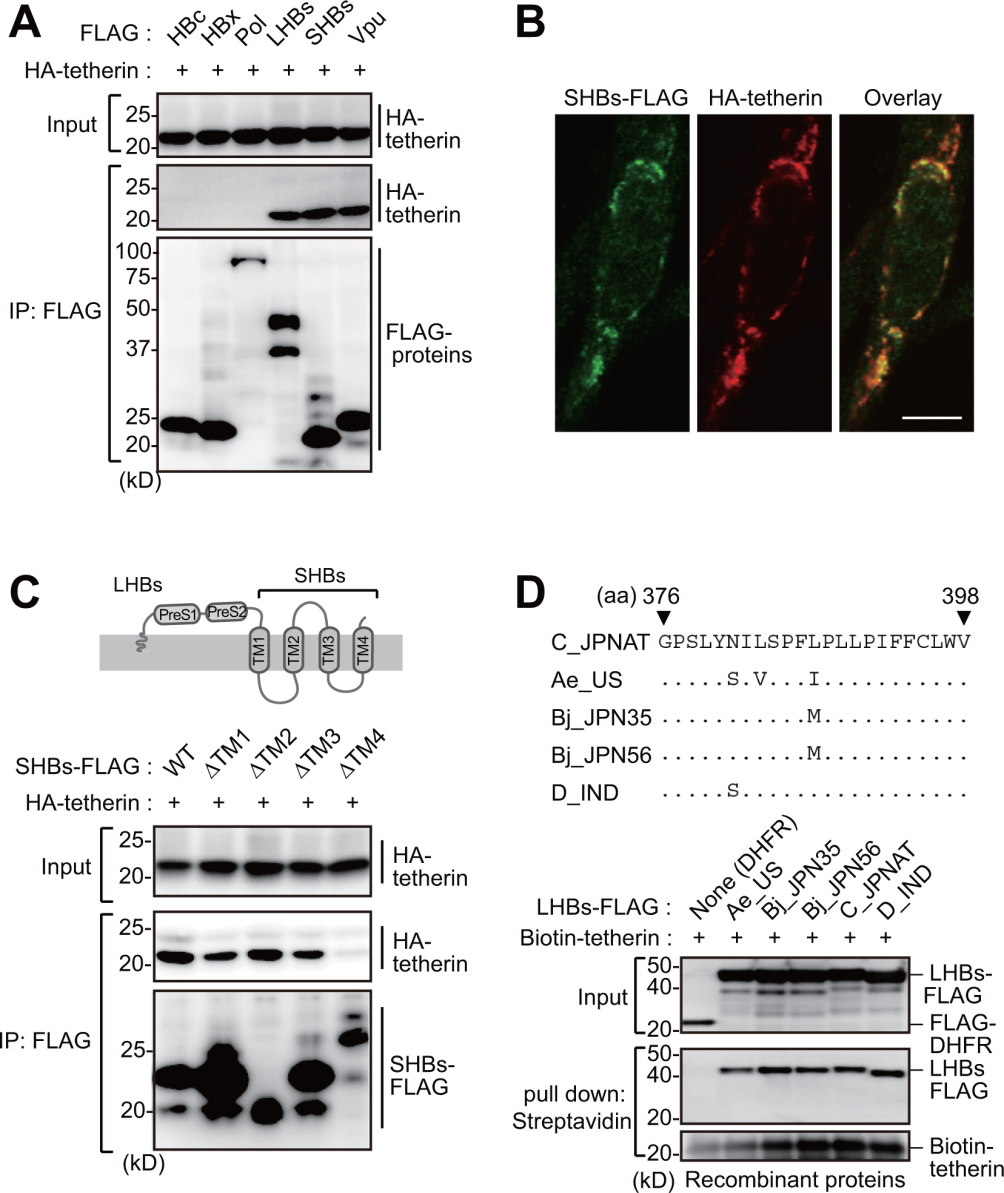
HBs binds tetherin via its fourth transmembrane domain **A.** HEK293 cells were cotransfected with plasmids encoding HA-tetherin and the indicated FLAG-tagged HBV proteins. Cell lysates were immunoprecipitated with anti-FLAG antibody and then analyzed by immunoblotting with either anti-HA or anti-FLAG antibody. Vpu (tetherin-interacting HIV protein) was used as a positive control. **B.** HepG2 cells were transfected with plasmids encoding SHBs-FLAG and HA-tetherin. After 24 h, the cells were fixed, permeabilized, and stained with anti-FLAG (green) and anti-HA (red), followed by confocal microscopic analysis. Scale bar, 10 μm. **C.** Schematic representation of the domain structure of HBs (top). HEK293 cells were transfected with WT SHBs-FLAG or its transmembrane domain-deficient mutants (ΔTM) together with HA-tetherin. Cell lysates were immunoprecipitated with anti-FLAG antibody, and the bound proteins were analyzed by immunoblotting with either anti-HA or anti-FLAG antibody (bottom). **D.** Alignment of the HBs TM4 sequence with the indicated HBV variants (top). Recombinant FLAG-tagged LHBs derived from the indicated HBV genotypes were mixed with recombinant biotinylated tetherin and then processed for the *in vitro* pull-down analysis with streptavidin sepharose beads. Captured proteins were analyzed by immunoblotting with either anti-FLAG antibody or horseradish peroxidase-conjugated streptavidin (bottom). DHFR (dihydrofolate reductase) was used as a negative control.

Hepatocytes have been reported to remain permissive for HBV infection regardless of the treatment of IFNα [16]. This is indicative of antagonistic properties of HBV against endogenous tetherin. To delineate this hypothesis, we assessed whether HBV-encoding proteins could interact with and inactivate tetherin. Immunoprecipitation analysis revealed that both large and small HBs (LHBs and SHBs) can interact with tetherin, but that no other viral protein (such as HBc, HBx and polymerase) could do so (Figure 2A). To verify the association between SHBs and tetherin in cells, we next examined the intracellular localization of both proteins by immunofluorescence confocal microscopy. Our results indicated that these proteins co-localize in the perinuclear region and cytoplasm (Figure 2B). We next attempted to identify the domain within SHBs that is responsible for the interaction with tetherin. Since SHBs has four transmembrane regions, we generated deletion mutants of these regions. Immunoprecipitation analysis with these SHB mutants indicated that the most C-terminal transmembrane domain (TM4) of SHBs is important for the binding of tetherin, since SHBs lacking TM4 (SHBsΔTM4) were not co-immunoprecipitated with tetherin (Figure 2C). Moreover, *in vitro* pull-down analysis using recombinant HBs (genotype A, B, C and D) and tetherin proteins synthesized in cell-free protein systems [17], clearly demonstrated that their interactions are evolutionally conserved across all of the HBV genotypes tested (Figure 2D).

### HBs inhibits the dimerization of tetherin to counteract its antiviral activity

**Figure 3:**
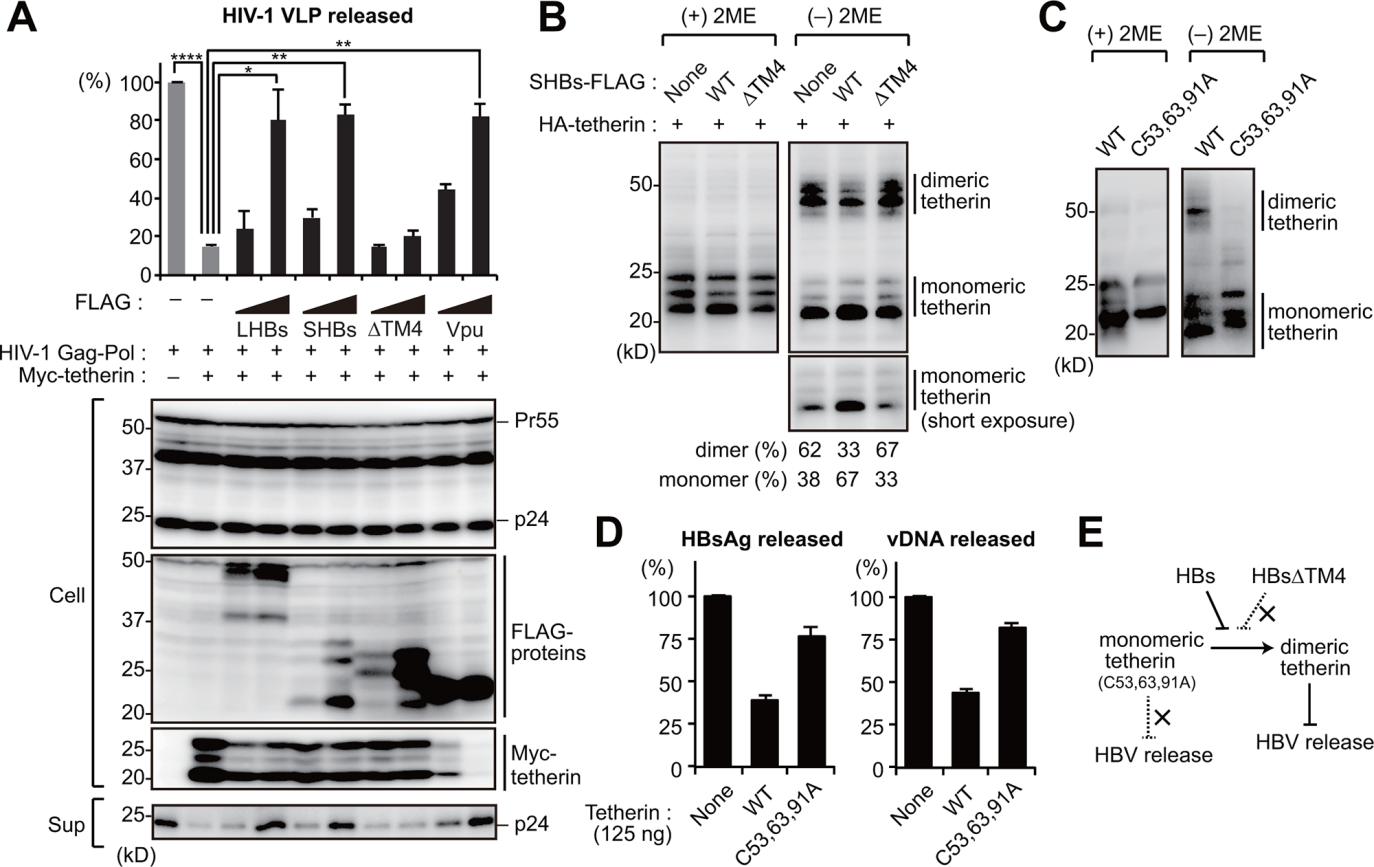
HBs inhibits the dimerization of tetherin to counteract its antiviral activity **A.** HIV-1 VLP release assay was performed in HepG2 cells transfected with the indicated vectors encoding HBs proteins (LHBs, SHBs and SHBsΔTM4) or Vpu (tetherin antagonist of HIV-1). At four days after transfection, the levels of p24 capsid protein in the culture supernatants were measured by ELISA (top). Blots below the bar graph show the detection of HIV-1 Gag (precursor Pr55 and p24 capsid), FLAG-tagged viral proteins and Myc-tetherin by immunoblotting (bottom). *P < 0.05; **P < 0.01; ****P < 0.0001. **B, C.** HEK293 cells were transfected with the vectors encoding HA-tetherin (100 ng) with or without SHBs expression plasmids (1 μg). Cell lysates were subjected to SDS-PAGE in the absence or presence of 2-mercaptoethanol (2ME), and analyzed by immunoblotting with anti-HA antibody. The numerical values below the blot indicate the amounts of dimer or monomer bands determined by densitometry. **D.** HepG2 cells were cotransfected with pUC19-C_JPNAT (2.5 μg) and vectors encoding WT or C53,63,91A tetherin (125 ng). At four days after transfection, the amounts of HBsAg and vDNA in the culture supernatants were measured. Note that 125 ng of WT tetherin can inhibit HBV release (see Figure 5B). **E.** Predicted model for the HBs-mediated counteraction of tetherin.

We next evaluated the antagonizing effect of HBs on the function of tetherin using a HIV-1 viral-like particle (VLP) model. In this model, HepG2 cells are cotransfected with vectors encoding HIV-1 Gag-Pol and tetherin together with either HBs (LHBs, SHBs and SHBsΔTM4) or HIV-1 Vpu (tetherin antagonist of HIV-1) as a positive control. As reported previously, the expression of tetherin restricted HIV-1 VLP release by approximately 10-fold in our ELISA experiments. This restriction was recovered by expression of LHBs and SHBs as well as Vpu. However, the SHBs mutant lacking tetherin binding site (SHBsΔTM4) failed to recover VLP release (Figure 3A). These results suggest that the direct interaction of HBs with tetherin via TM4 domain might be essential for the anti-tetherin function of HBs. Although Vpu has been reported to decrease the expression of tetherin in cells [18, 19], we did not observe this same effect in case of HBs (Figure 3A).

Since HBs was found to interact directly with tetherin (Figure 2D), we hypothesized that it might interfere with the dimer formation of tetherin. As previously reported [20, 21], wild-type (WT) tetherin is detectable as a dimer (50 kD) under non-reducing conditions (Figure 3B). Interestingly, we found in our current experiments that HBs, but not HBsΔTM4, increased the monomeric tetherin level (Figure 3B), suggesting that HBs counteracts the antiviral activity of tetherin by inhibiting dimerization. Consistently, the overexpression of a dimerization-defect tetherin mutant (C53, 63, 91A) [20] had no effects on HBV release (Figure 3C, 3D). Collectively, these results suggest that HBs can bind and antagonize tetherin by inhibiting the functional dimerization of tetherin (Figure 3E).

### The transmembrane domain of tetherin is responsible for HBs binding

**Figure 4:**
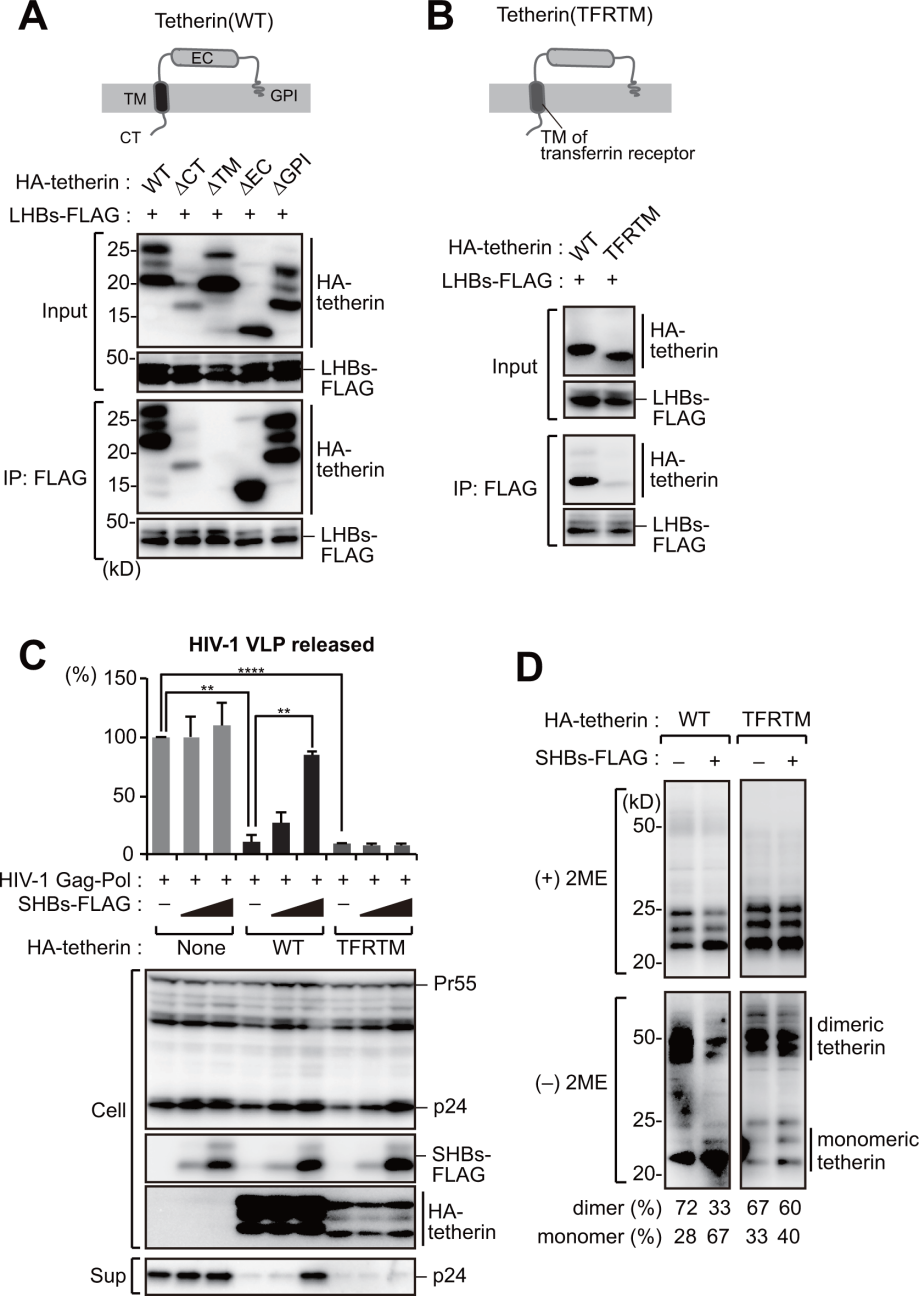
The transmembrane domain of tetherin is responsible for HBs binding **A.** Schematic representation of the domain structure of tetherin (top). HEK293 cells were transfected with SHBs-FLAG and cotransfected with WT tetherin or its domain mutants (CT: cytoplasmic tail; TM: transmembrane; EC: extracellular domain, GPI: glycosylphosphatidylinositol anchor). Cell lysates were then immunoprecipitated with anti-FLAG antibody, and the bound proteins were analyzed by immunoblotting with either anti-HA or anti-FLAG antibody (bottom). **B.** Schematic representation of a substitution mutant of tetherin harboring a TM domain derived from transferrin receptor (TFRTM) (top). WT tetherin and its TFRTM mutant were subjected to the immunoprecipitation analysis as in (A) (bottom). **C.** HIV-1 VLP release assay in HepG2 cells transduced with WT tetherin and its TFRTM mutant together with the indicated vectors, as in Figure 3A. **P < 0.01; ***P < 0.001; ****P < 0.0001. **D.** Detection of tetherin dimerization in HEK293 cells transfected with the indicated vectors encoding HBs and HA-tetherin, as in Figure 3B. The numerical values below the blot indicate the amounts of dimer or monomer bands determined by densitometry.

We next mapped the binding domain within tetherin that interacts with HBs. Tetherin consists of an N-terminal cytoplasmic (CT) domain, single transmembrane (TM) domain, extracellular (EC) domain and a C-terminal glycosylphosphatidylinositol (GPI) anchor (Figure 4A). Tetherin mutants lacking these domains were generated and subjected to immunoprecipitation analysis. All of these tetherin mutants except for ΔTM efficiently interacted with HBs (Figure 4A), indicating that the tetherin transmembrane domain is responsible for the HBs binding. To further confirm the possibility that HBs antagonizes tetherin through TM-TM associations, we substituted the TM domain of tetherin with the corresponding domain of transferrin receptor, hereafter referred to as TFRTM tetherin (Figure 4B). As expected, TFRTM tetherin showed almost no interaction with HBs in our immunoprecipitation analysis (Figure 4B). A HIV-1 VLP release assay demonstrated that the antiviral function of TFRTM tetherin were comparable with WT tetherin (Figure 4C), as reported previously [22]. However, whereas the antiviral function of WT tetherin was inhibited by HBs, that of TFRTM tetherin was not (Figure 4C). Consistently, the dimerization of TFRTM tetherin was also not abrogated by HBs expression (Figure 4D).

### HBs-resistant chimeric tetherin efficiently restricts HBV release

**Figure 5:**
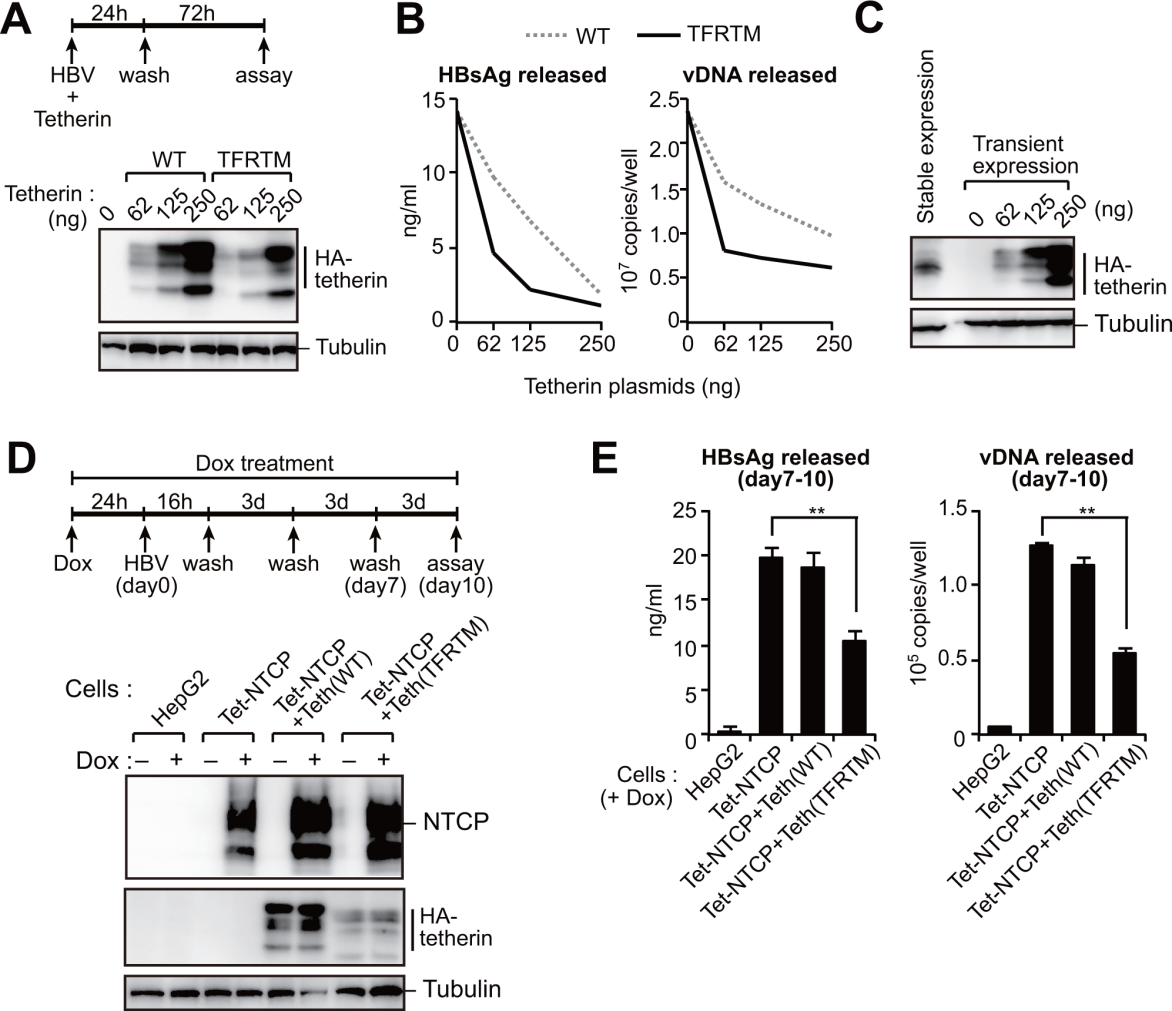
HBs-resistant chimeric tetherin efficiently restricts HBV release **A, B.** HepG2 cells were cotransfected with pUC19-C_JPNAT (2.5 μg) and the indicated amounts of expression vector encoding WT or TFRTM tetherin. Four days after transfection, the indicated protein expression in cells was detected by immunoblotting (A). The HBsAg or vDNA levels in the culture supernatants were measured by ELISA or real-time PCR, respectively (B). Data are representative of three experiments. **C.** Comparison of tetherin expression in stably- and transiently-expressing HepG2 cells. **D, E.** Indicated cells were infected with HBV in the presence or absence of Dox. At 11 days after infection, the indicated protein expression in cells was detected by immunoblotting (C). The amounts of HBsAg and vDNA in the culture supernatants were measured by ELISA and real-time PCR, respectively (D). **P < 0.01.

We next examined the effect of HBs-resistant tetherin on HBV release. Consistent with the data from our HIV-1 VLP assay (Figure 4C), TFRTM tetherin exhibited more potency for inhibiting HBV release and was effective even at relatively lower amounts (i.e, 62 ng/well) (Figure 5A, 5B). Recent studies have demonstrated that the stable expression of NTCP (Na^+^ taurocholate cotransporting polypeptide) in HepG2 cells allows for HBV entry, viral protein synthesis, and subsequent production of progeny virions [23–25]. We thus generated HepG2 cells harboring a tetracycline-inducible NTCP gene, referred to hereafter as HepG2-Tet-NTCP. We also produced HepG2-Tet-NTCP cells that stably expressed tetherin at a lower level (Figure 5C). We confirmed that these cell lines expressed an equivalent level of NTCP in the presence of doxycycline (Figure 5D). These cells were infected with HBV for 10 days and the virus levels in the culture supernatants were then measured. Interestingly, HBV particle release from HepG2-Tet-NTCP cells was found to be comparable to that from those additionally expressing WT tetherin (Figure 5E), suggesting that WT tetherin failed to inhibit HBV release due to a HBs-mediated counteracting mechanism. In contrast, HepG2-Tet-NTCP cells expressing TFRTM tetherin demonstrated significantly reduced levels of progeny virions in the culture supernatant (Figure 5E). These results suggested that even at lower levels, TFRTM tetherin can effectively restrict HBV release.

### TFRTM tetherin inhibits virus-induced cytotoxicity in iPSC-derived hepatocytes

**Figure 6:**
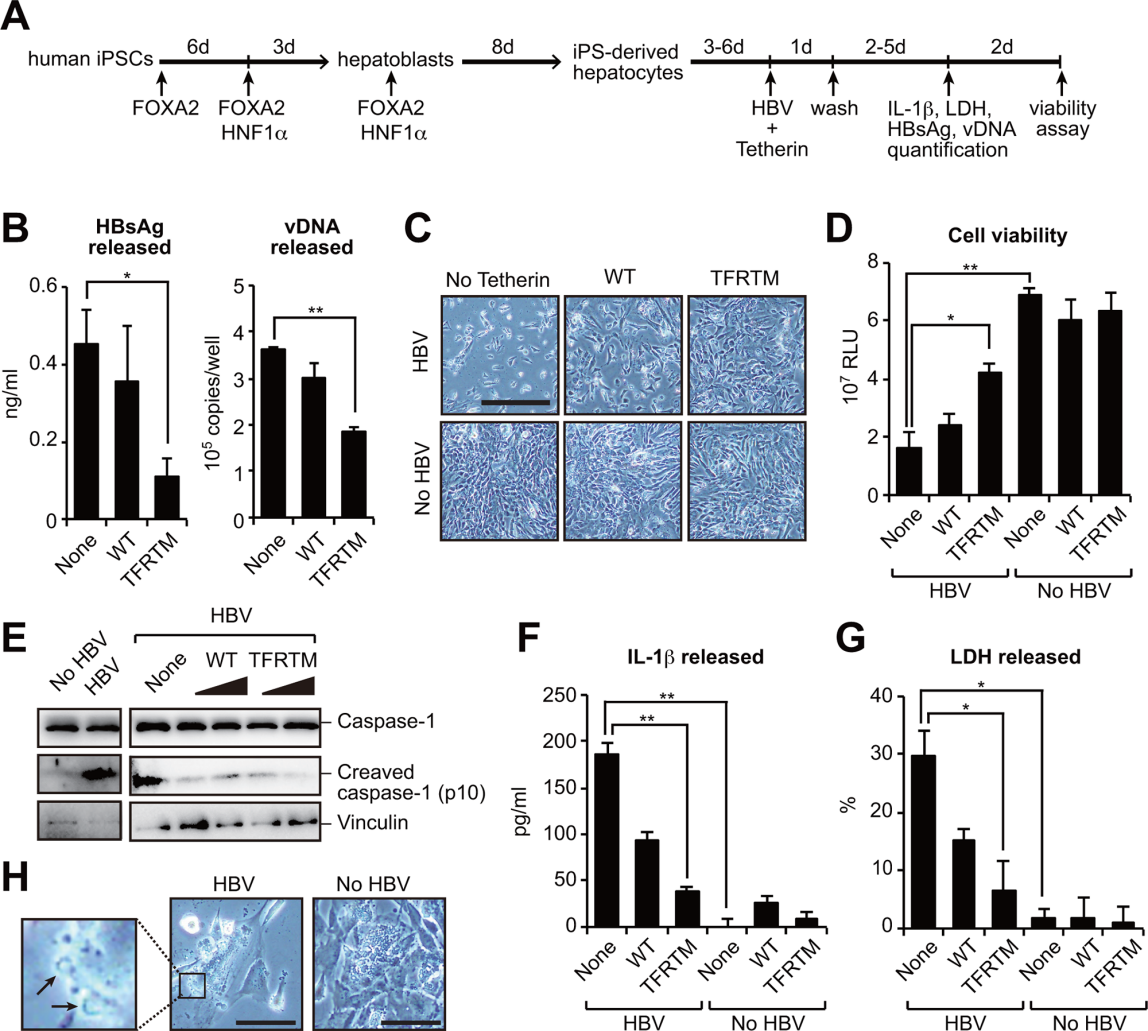
Transduction of TFRTM tetherin inhibits virus-induced cytotoxicity in iPSC-derived hepatocytes **A, B.** Hepatocyte-like cells were differentiated from human induced pluripotent stem cells (iPSCs) according to a previously described method [42] (A). iPSC-derived hepatocytes were cotransfected with pUC19-C_JPNAT and vectors encoding WT or TFRTM tetherin. Four days after transfection, the amounts of HBsAg and vDNA in the culture supernatants were measured using ELISA and real-time PCR, respectively (B). *P < 0.05; **P < 0.01. **C, D.** Microscopic images (C) and cell viability (D) of indicated hepatocytes at 7 days after transfection. Scale bar, 200 μm. *P < 0.05; **P < 0.01. **E–G.** Detection of cleaved caspase-1 in cell lysates (E) and secreted IL-1β (F) and LDH (G) in the culture supernatants of indicated hepatocytes at 4 days after transfection. *P < 0.05; **P < 0.01. **H.** Plasma membrane raptures (black arrow) were mainly observed in HBV-transduced hepatocytes (at 3 days after transfection). Scale bar, 50 μm.

The aforementioned results demonstrated that TFRTM tetherin has potent anti-HBV activity. Hence, a therapeutic strategy to utilize the TFRTM tetherin would have potential benefits. The recent development of induced pluripotent stem cell (iPSC) technology enabled us to utilize iPSC-derived hepatocytes in liver diseases including virus-induced hepatitis. We thus cotransfected human iPSC-derived hepatocytes with either WT or TFRTM tetherin and a HBV molecular clone to assess the inhibitory effect of TFRTM tetherin on HBV release (Figure 6A). Compared with WT tetherin, TFRTM tetherin strongly inhibited the release of HBV (Figure 6B). Although the aberrant expression of HBV caused prominent cytotoxicity in iPSC-derived hepatocytes, this was completely reverted by TFRTM tetherin (Figure 6C, 6D), suggesting its cytoprotective activity against HBV.

Several previous reports have demonstrated that the transduction of HBV DNA into primary hepatocytes induced cell death, possibly by apoptosis [26, 27]. However, we could not detect cleaved caspase-3, an apoptotic marker, in HBV-transduced iPSC-derived hepatocytes (data not shown) but instead detected the inflammasome-mediated pyroptosis markers cleaved caspase-1 and interleukin (IL)-1β [28] in cell lysate and culture supernatant, respectively (Figure 6E, 6F). Caspase-1 activation induces cell swelling and the release of cytosolic contents, including lactose dehydrogenase (LDH) [29]. Indeed, we detected LDH secretion (Figure 6G) and the formation of membrane rupture in HBV-transduced hepatocytes (Figure 6H). Notably, TFRTM tetherin decreased the levels of these pyroptosis markers (Figure 6E, 6F, 6G). Taken together, our current data provide the proof of concept that the use of TFRTM tetherin is a viable strategy for cell-mediated anti-HBV therapy in the context of inhibiting viral release and virus-induced cytopathic effects.

## DISCUSSION

In our current study, we decipher the molecular link between HBV and tetherin during the course of HBV infection as part of an IFN-induced antiviral response. We find that tetherin can block HBV release, but the HBV surface protein, HBs, can antagonize the antiviral activity of tetherin. This scenario strongly supports the “offensive and defensive battle” theory between host and virus [30], in which both players can be reciprocally suppressed in accordance with the quantity and/or capability ratio of tetherin versus HBs. When HBs is more predominant than tetherin (e.g. during the acute infection phase), HBs can inactivate the antiviral function of tetherin via direct interaction through the TM4 domain. Conversely, if tetherin predominates, it can restrict the release of HBV.

Indeed, many viral proteins can inactivate tetherin in multiple ways [6]. For example, Ebola virus Glycoprotein (GP) counteracts tetherin without inducing its degradation. Although the mechanism of this is not known, GP needs to bind tetherin directly to antagonize its function [9]. We found in our current study that HBs can inhibit the dimerization of tetherin, which is an essential process for anti-HBV activity. This is the first evidence of a mechanistic link between HBs and tetherin. Additionally, a recent study has indicated that in plasmacytoid dendritic cells, HBs abrogates the TLR7/9-induced innate immune reaction towards IFNα gene transcription [31], suggesting that as an upstream effector, HBs may downregulate the expression of ISGs itself *in vivo*.

The role of intracellular restriction factors in HBV infection as a part of the type I IFN pathway has not been well studied. Several ISGs, including APOBEC3G and IDO, have been shown to act as putative anti-HBV factors in *in vitro* culture models [32, 33]. However, for unknown reasons, the response rate of IFNα-treated hepatitis B patients was found previously to be very poor regardless of the induction of ISGs [34]. This discrepancy between *in vitro* and *in vivo* effects may be partly due to the stoichiometric balance between ISGs and its viral countermeasure (such as tetherin and HBs). Indeed, our current study findings demonstrated the effect of IFN-induced tetherin on viral release to be relatively weak, but that an overabundance of HBs-resistant chimeric tetherin strongly inhibited HBV release in comparison with WT tetherin. Hence, the development of new methods for transducing HBs-resistant tetherin into hepatocytes could prove to be a unique strategy to efficiently suppress HBV replication. In this regard, our current study has demonstrated that the transduction of HBs-resistant tetherin into iPSC-derived hepatocytes confers potent anti-HBV activity and protects the cells from HBV-induced cytotoxicity. This therapeutic strategy would be more practicable by utilizing recently developed genome editing and stem cell technologies.

In our current analyses, we unexpectedly found that HBs-resistant tetherin strongly inhibited HBV-triggered cell death, accompanied by caspase-1 activation and IL-1β secretion. These are markers of “pyroptosis”—a proinflammatory cell death process that eliminates the infected cell [35]. During viral infection, viral DNA/RNA recognized by pattern recognition receptors (PRRs) in host cells can promote the formation of inflammasomes, resulting in the activation of caspase-1. This induces pyroptosis together with the secretion of proinflammatory cytokines IL-1β and IL-18 [35]. Several previous reports have demonstrated that AIM2, a cytosolic PRR, enhances immunopathology during HBV infection [36, 37]. Tetherin may block the AIM2-dependent signaling pathway via unknown mechanisms. Alternatively, in the absence of tetherin, vast quantities of virions (including Dane particles, HBsAg or HBcAg subviral particles) released from HBV-producing cells may trigger pyroptosis. Indeed, HBcAg has been shown to induce the secretion of bioactive IL-18, another marker of pyroptosis, an event which is blocked by caspase-1 inhibitor [38]. Although the mechanisms underlying how PRRs sense HBcAg are not yet known, HBV-mediated pyroptosis can be activated by HBcAg invasion. Nonetheless, blockage of HBV-associated hepatic injury may be a therapeutic strategy for the treatment of chronic hepatitis B. Further studies should identify the involvement of inflammasome-mediated hepatocyte death in HBV infection.

In conclusion, tetherin plays an important role in intracellular antiviral immunity during the course of HBV infection. Strategies to augment the antiviral activity of tetherin by impeding tetherin-HBs interactions may be a viable therapeutic intervention against HBV. Moreover, a better understanding of how HBV evades IFN-induced host immune response during infection may help to develop more effective vaccines.

## MATERIALS AND METHODS

### Plasmids

The HBV molecular clone pUC19-C_JPNAT (genotype C) has been described previously [39]. Tetherin, HIV-1 Gag-Pol and Vpu expression vectors have also been described previously [40, 41]. The tetherin mutants, ΔCT (Δ2–21 aa), ΔTM (Δ22–44 aa), ΔEC (Δ45–160 aa), ΔGPI (1–160 aa) and C53,63,91A, were generated using PCR-based molecular cloning procedures. To generate HBs-resistant tetherin mutants, the TM domain of tetherin was replaced with the corresponding domain of human transferrin receptor (63–88 aa). HBV LHBs, SHBs, HBc, HBx and polymerase cDNAs were synthesized or amplified from pUC19-C_JPNAT with the appropriate primer pairs, followed by subcloning into the pCMV-3xFLAG vector (Sigma-Aldrich, St Louis, MO). The HBs derivatives, ΔTM1 (Δ180–201 aa), ΔTM2 (Δ254–273 aa), ΔTM3 (Δ344–366 aa) and ΔTM4 (Δ376–398 aa), were constructed using PCR-based mutagenesis. For viral vector production, NTCP and tetherin cDNAs were inserted into the packaging vector pRetroX-TRE3G (Clontech, Palo Alto, CA) and CSII-CMV-MCS-IRES2-Bsd (RIKEN BRC, Ibaraki, Japan), respectively.

### Cells

HEK293 cells (ATCC) were cultured in DMEM containing 10% FBS. HepG2 cells (ATCC) were maintained on collagen-coated dishes with DMEM/F-12, GlutaMAX (Life Technologies, Gaithersburg, MD) supplemented with 10% fetal bovine serum, 10 mM HEPES and 5 μg/ml insulin. To generate HepG2-Tet-NTCP cells, the parental HepG2 Tet-On Advanced cells (Clontech) were infected with a RetroX-based retroviral vector encoding the NTCP gene, and selected with 1 μg/ml puromycin. Derivatives of HepG2-Tet-NTCP cells stably expressing tetherin were generated by transduction with CSII-based lentiviral vectors expressing WT or TFRTM tetherin, followed by selection in 10 μg/ml blasticidin. Primary human hepatocytes were purchased from Phoenixbio (Hiroshima, Japan). Human iPSC-derived hepatocytes were generated by ReproCELL (Kanagawa, Japan) according to a previously described procedure [42]. Retroviral vectors were prepared from 293-GP2 cells (Clontech) in 6-cm dishes cotransfected with the RetroX packaging vector (3 μg) and VSV-G expression plasmids (1 μg) in accordance with the manufacturer's instructions. For the production of lentiviral vectors, 293T cells in 6-cm dishes were cotransfected with the CSII packaging vector (6 μg), HIV-1 Gag-Pol (3.5 μg) and VSV-G expression plasmids (3.5 μg). Culture supernatants containing viral vectors were collected at 48 h after transfection, filtered through a 0.45-μm filter (Merck Millipore, Billerica, MA).

### Transfection-based HBV release assays

HepG2 cells in 6-well plates were cotransfected with pUC19-C_JPNAT (2.5 μg) and a tetherin expression vector (62–250 ng) using GenJet *in vitro* DNA transfection reagent (SignaGen, Rockville, MD) or Lipofectamine 3000 (Life Technologies) in accordance with the manufacturer's instructions. At 3 days post-transfection, culture supernatants were cleared of cell debris by centrifugation at 3,000 rpm for 3 min. The HBsAg quantities in each sample were then measured using a HBsAg ELISA kit (Alpha Diagnostic, San Antonio, TX). Cells were suspended with lysis buffer (100 mM Tris-HCl (pH 8.0), 0.2% NP-40) and clarified by centrifugation at 13,000 rpm for 1 min. To remove the plasmid-derived DNA, the cell lysates or culture supernatants (250 μl) were were digested with 200 μg/ml DNase I, 100 μg/ml RNase A and 6 mM MgOAc for 2 h at 37°C, and centrifuged at 13,000 rpm for 1 min. The supernatants were then mixed with a solution of 10 mM EDTA, 1% SDS, 100 mM NaCl and 200 μg/ml proteinase K (Roche Diagnostics, Basel, Switzerland), and incubated at 55°C for 1 h. These samples were extracted with phenol/chloroform, precipitated with ethanol and dissolved in TE buffer (10 mM Tris-HCl (pH 8.0), 1 mM EDTA). Viral DNA was then quantified by real-time PCR using SYBR Premix Ex Taq II (Takara, Shiga, Japan) and a CFX-96 system instrument (Bio-Rad, Hercules, CA). The primer pair used was 5′-gagtgtggattcgcactcc-3′ (HBV2270F) and 5′-gaggcgagggagttcttct-3′ (HBV2392R) [23].

In the siRNA and IFNα experiments (Figure 1), one day prior to the transfection of the HBV molecular clone, cells were transduced with 60 pmol tetherin-specific siRNA (HSS101113 and HSS101114, Life Technologies) or control siRNA using Lipofectamine RNAiMAX (Life Technologies). Cells were then pre-treated with 1000 U/ml IFNα (Sigma-Aldrich) for 3 h before transfection. At 24 h after transfection of pUC19-C_JPNAT (2.5 μg), cells were washed twice and then additionally cultured for three days with or without IFNα, followed by quantification of HBsAg and vDNA, as described above.

### HBV preparation and infection

HBV stocks were derived from the supernatants of HepG2.2.15 cells, which were stably transfected with a complete HBV genome (genotype D). The collected supernatants were filtered through a 0.45-μm filter (Merck Millipore), and concentrated using PEG virus precipitation kit (BioVision, Milpitas, CA). HepG2-Tet-NTCP or its derivative cells in 24-well plates were infected with HBV (5000 GEq/cell) with or without 5 μg/ml Dox. The culture supernatants were then harvested and subjected to quantification of HBsAg and vDNA, as described above.

### Immunoprecipitation, *in vitro* pull-down and immunoblotting

Immunoprecipitation were performed as previously described [43, 44]. *In vitro* streptavidin pull-down analysis with recombinant proteins were also performed as previously described [43]. Briefly, biotinylated tetherin was incubated with SHBs-FLAG at 26°C for 2 h before being co-incubated with streptavidin-Sepharose beads (GE Healthcare, Little Chalfont, UK) at 4°C for 3 h. Bound proteins were analyzed by immunoblotting as follows. Samples in SDS loading buffer (with or without 2-mercaptoethanol) were loaded onto 10% or 15% gels and blotted onto PVDF membranes (Merck Millipore). Membranes were probed with primary antibodies and horseradish peroxidase-conjugated secondary antibodies (GE Healthcare). The antibodies used in this study were as follows: anti-tetherin (a gift from Chugai Pharmaceuticals), anti-α-tubulin (Sigma-Aldrich), anti-HA (Roche), anti-FLAG (Sigma-Aldrich), anti-Myc (Cell Signaling Technology, Danvers, MA), anti-p24 (NIH AIDS Reagent Program), anti-NTCP (Sigma-Aldrich), anti-vinculin (Sigma-Aldrich) and anti-caspase1 (Abcam, Cambridge, MA). The proteins detected were visualized on a FluorChem digital imaging system (Alpha Innotech, San Leanardo, CA) and the band intensities were quantified with NIH ImageJ software.

### HIV-1 VLP release assays

HepG2 cells in 12-well plates were transfected with vectors encoding HIV-1 Gag-Pol (200 ng), tetherin (100 ng) and either HBs (200 or 1000 ng) or Vpu (100 or 500 ng). At four days after transfection, culture supernatants were harvested and clarified, and p24 antigens were measured with an HIV-1 p24 ELISA kit (Zepto Metrix, Buffalo, NY). For immunoblotting analysis, the virus-containing supernatants was layered onto 20% sucrose in PBS and centrifuged at 20,000 g for 2 h. Cell and virion lysates were then subjected to immunoblotting analysis as described above.

### Measurements of secreted LDH and IL-1β, and cell viability

Human iPSC-derived hepatocytes in 12-well plates were transfected with of pUC19-C_JPNAT (1 μg) together with vectors expressing tetherin (125 or 250 ng) and GFP (200 ng, as a transfection control) using Lipofectamine 3000 (Life Technologies). At 2-5 days after transfection, the culture supernatants were collected and secreted LDH and IL-1β were assayed using an LDH cytotoxicity detection kit (Roche) and IL-1β ELISA kit (R&D systems, Minneapolis, MN), respectively. At day 4-7 after transfection, cell viability was determined by using Cell Titer-Glo (Promega, Madison, WI).

### Immunofluorescence

One day before transfection, cells were seeded onto collagen-coated glass cover slip. At 48 h post-transfection, the cells were fixed with 4% paraformaldehyde and stained as described previously [41]. Alexa Fluor-conjugated secondary antibodies (Life Technologies) were used to detect signals. Microscopic imaging was performed with an FV1000-D confocal laser scanning microscope (Olympus, Tokyo, Japan).

### Statistical analysis

All graphs present the means and SDs. The statistical significance of differences between two groups was tested using a two-tailed unpaired *t* test with Prism 6 software (GraphPad, La Jolla, CA). A *P* value of <0.05 was considered statistically significant.
